# Dentin Bonding and SEM Analysis of a New Experimental Universal Adhesive System Containing a Dendrimer

**DOI:** 10.3390/polym12020461

**Published:** 2020-02-17

**Authors:** Joana Vasconcelos e Cruz, Mário Polido, José Brito, Luisa L. Gonçalves

**Affiliations:** 1 Instituto de Ciências Biomédicas de Abel Salazar (ICBAS), Universidade do Porto, Rua de Jorge Viterbo Ferreira 228, 4050-313 Porto, Portugal; joanavcruz@gmail.com; 2 Instituto Universitário Egas Moniz (IUEM), Campus Universitário, Quinta da Granja, Monte de Caparica, 2829-511 Caparica, Portugal; mpolido@egasmoniz.edu.pt (M.P.); britojaa@hotmail.com (J.B.); 3 Centro de Investigação Interdisciplinar Egas Moniz (CiiEM), Campus Universitário, Quinta da Granja, Monte de Caparica, 2829-511 Caparica, Portugal

**Keywords:** dendrimers, dental adhesives, G-IEMA, methacrylate, dentin bonding, SEM analysis

## Abstract

Due to their polymerization characteristics, hyper-branched dendrimers have lately shown to be promising candidates for use in dental materials. In this study, a new dental adhesive system was prepared, using a dendrimer derived from 2-isocyanatoethyl methacrylate (G-IEMA), and its adhesive properties were investigated. The exposed dentin was treated with four universal adhesives (UAs): SBU (Scotchbond Universal^™^), FUT (Futurabond M+^™^), AE1 (experimental adhesive with Bis-GMA) and AE2 (experimental adhesive with G-IEMA), using Etch & Rinse (ER) or Self Etch (SE) protocols. Composite build-ups were prepared and stored for 24 h at 37 °C in distilled water. Composite/dentin beams were prepared with cross-sectional areas of 1 ± 0.3 mm^2^ and µTBS (Micro-tensile bond strength) test was performed at 0.5 mm/min. Failures modes were evaluated by stereomicroscopy, and bonding interfaces were observed by scanning electron microscopy (SEM). Statistical analysis of µTBS data was performed using General Linear (GLM) and Linear Mixed Models (LMM). The effect of adhesive type on µTBS was significant (*p* = 0.010), with AE1 presenting significantly higher µTBS than SBU (*p* = 0.019). No other differences between adhesives were observed. ER showed significantly better results than SE (*p* = 0.019), and no significant interactions between the adhesives and protocols were determined. Results obtained so far pinpoint the emergence of a new paradigm in the dental materials field, as G-IEMA can be used successfully as an alternative to Bis-GMA.

## 1. Introduction

Dentin bonding remains a challenge in clinical practice [1,2,3]. The high protein and water content of dentin makes it a very heterogeneous and dynamic substrate, increasing the difficulty of the bonding procedure [4]. Dentin bonding is obtained by mechanical retention of the adhesive monomers within the hybrid layer (HL) and the dentin tubules. The HL results from the infiltration and polymerization of the adhesive monomers between the collagen fibers exposed due to partial demineralization of dentin, sometimes enhanced by chemical adhesion [5,6]. The stability of the HL is critical for bonding efficacy; however, the main reason for failure has been attributed to the presence of water and acidic components that enhance enzymatic and hydrolytic degradation of newly formed HL [6].

Determining the dentin wetness for bonding stability is quite difficult. On one hand, water is required for the expansion of the collagen network and diffusion of the adhesive monomers into the dentin. On the other, the excess water causes hydrolysis of the hydrophilic components of the bonding interface, leading to adhesive failure [3,4,7].

There are two strategies for promoting dentin adhesion—the etch-and-rinse (ER) and the self-etch (SE) protocols, both presenting advantages and disadvantages [8]. The ER protocol creates a greater dentin demineralization depth than SE, allowing the adhesive infiltration to form a thick hybrid layer. However, this technique presents a higher risk of dentin dehydration and the collapse of collagen fibers [9]. With SE, demineralization degree is more superficial, and the hybrid layer is thinner. Nevertheless, this protocol allows for better dentin wetness control and maintains higher calcium content availability for chemical bonding with specific functional monomers, which are included in the chemical composition of the adhesives [7,10,11].

Universal adhesives (UAs), or multi-mode adhesives, are a versatile adhesive family designed under the “all-in-one” concept, allowing either ER or SE application depending on the clinical situation or dentists’ choices [12,13]. 

The composition of UAs comprises of a mixture of hydrophilic and hydrophobic monomers, polymerization initiators, solvents, stabilizers and filler particles [8,12]. Additionally, most UAs also contain specific carboxylate and/or phosphate monomers, like 10-MDP (10-Methacryloyloxydecyl-dihydrogen phosphate), that bond ionically to calcium from hydroxyapatite [14]. This acidic and functional monomer contributes greatly to strengthen the micromechanical adhesive bonding to dentin when the SE protocol is applied, producing chemical bond adhesion [15]. Clinically, the stability of this type of 10-MDP-mediated chemical bonding has resulted in an excellent 13-year retention rate for the two-step 10-MDP-based Clearfil SE Bond (Kuraray Noritake Dental, Okayama, Japan) [14]. 

Despite the benefits of the simplified UAs, they still present limitations. In vitro and clinical performances seem to be dependent on their chemical composition [9], with the presence of water being one of the main problems. Water is required for the ionization of the acidic monomers, enabling them to interact with dentin and enamel. Residual water causes hydrolytic degradation to both monomers and collagen, which is enhanced by the acidic pH of the monomer [14].

Another composition related concern is the use of Bis-GMA. Its high viscosity makes it difficult to diffuse into dentin tissue. To reduce viscosity, Bis-GMA is always diluted with other monomers, such as the more hydrophilic triethylene-glycol-dimethacrylate (TEGDMA) and hydroxyethyl-methacrylate (HEMA), which increases the water content and polymerization shrinkage [10,16]. Additionally, Bis-GMA contains bisphenol A (BPA), whose release may have a negative impact on human health. BPA has been described as a cytotoxic agent and may be responsible for estrogenic activity [17], acting like an endocrine disruptor [18,19]. Some authors claim that the use of restorative materials with Bis-GMA might increase human exposure to BPA [19]. However, some others defend that Bis-GMA does not hydrolyze into BPA, due to its chemical structure which prevents hydrolysis at ester linkage [18]. Nevertheless, this current and controversial issue led many manufacturers to seek alternatives for the removal of Bis-GMA from dental material’s formulations [17,20].

Considering the abovementioned problems affecting the use of adhesives, it seems that one of the major current challenges is the development of new dental adhesive systems and techniques that reduce the hydrolysis of hybrid layers, improving bonding longevity and, from a biological point of view, without bisphenol A. 

More hydrophobic adhesives with low polymerization shrinkage, high double bond conversion and high penetration ability were considered the main characteristics to take into account to improve bonding efficacy [21]. Consequently, dendritic macromers have recently been introduced in dental materials. Their polymerization characteristics were claimed to improve the double bond conversion [20]. Published studies showed that the use of such monomers, such as G-IEMA (dendrimer of generation (2) derived from 2-isocyanatoethyl methacrylate), in restorative materials could be promising, as it enables the preparation of more hydrophobic materials with better physical and mechanical properties. Due to their polymerization characteristics, hyper-branched macromers are potential candidates to be used in dental materials, as it has the advantage of not containing Bisphenol A in its composition [20].

To the best of the authors’ knowledge, G-IEMA has never been used in dental adhesives systems. The first promising results of the incorporation of G-IEMA in a dental adhesive, published by the authors [22], revealed that the replacement of Bis-GMA by the aforementioned dendrimer presented good physicochemical properties, such as Double Bond Conversion (DBC), Polymerization Shrinkage (PS), Water Sorption (WS) and Solubility (SL), indicating that G-IEMA might be a good monomer for use in the preparation of Bis-GMA-free adhesive systems. Based on the results obtained so far, the replacement of Bis-GMA by G-IEMA might produce a Bis-GMA-free UA system that possesses the same dentin bond strength, regardless of the applied protocol. Thus, this study aimed to investigate the dentin adhesion efficacy, using two experimental UA systems: an experimental adhesive formulated with the traditional Bis-GMA, and another with an in-house synthesized dendrimer (G-IEMA). 

## 2. Materials and Methods 

### 2.1. Formulation of the Experimental Adhesives

Based on the disclosed composition of two commercial adhesives, Scotchbond Universal (3M ESPE, St. Paul, MN, USA) and Futurabond M+ (VOCO GmbH, Cuxhaven, Germany), two universal adhesives were formulated (AE1 and AE2). AE1 was prepared by mixing urethane-dimethacrylate (UDMA; 2-[[3,5,5-trimethyl-6-[2-(2-methylprop-2-enoyloxy)ethoxycarbonylamino]hexyl] carbamoyl-oxy]ethyl-2-methylprop-2-enoate), bisphenol-A-diglycidyl-dimethacrylate (Bis-GMA; [2-hydroxy-3-[4-[2-[4-[2-hydroxy-3-(2-methylprop-2-enoyloxy)propoxy]phenyl]propan-2-yl]phenol-xy]propyl],2-methylprop-2-enoate), triethylene-glycol-dimethacrylate (TEGDMA; 2-[2-[2-(2-methylprop-2-enoyloxy)ethoxy]ethoxy]ethyl-2-methylprop-2-enoate), hydroxyethyl-methacrylate (HEMA; 2-hydroxyethyl-2-methylprop-2-enoate), deionized water, ethanol, camphoroquinone (CQ; 1,7,7-trimethylbicyclo[2.2.1]heptane-2,3-dione) and 10-methacryloyloxy-decyl-dihydrogen phosphate (10-MDP). All reagents were purchased from Sigma Aldrich, Madrid, Spain, except 10-MDP, which was acquired from Watson International Ltd., Kunshan City, China.

For the formulation of AE2, G-IEMA was first synthesized following the protocol of Yu et al. (2014) [20] with minor changes, as described in detail in [22]. The isolated faction, corresponding to G-IEMA, presented the following more relevant spectroscopic features: FTIR (Fourier Transform infrared spectroscopy), ν(cm^−1^): 3355 ν(N–H, amide), 2956 ν(C–H, aliphatic), 1713 ν(C=O, ester), 1636 ν(C=C, aliphatic/alkene), 1544 δ (N–H, amide), 1453 δ(CH, alkene), 1158 ν(C–O, ester), and 942 δ(CH, aliphatic-alkene). ^1^H-NMR (proton nuclear magnetic resonance): 6.13 (s, 8H, trans, CH_2_=CH(CH_3_)CO); 5.53 (s, 8H, cis, CH_2_=C(CH_3_)CO); 4.16 (t, 16H, CH_2_CH_2_OCOC(CH_3_)=CH_2_); 4.07 (t, 16H, NCH_2_CH_2_OCONH); 3.67 (s, 8H, CCH_2_O–); 3.50 (t, 16H, HNCH_2_CH_2_OOC); 3.29 (s, 8H, CCH_2_OCO); 2.77 (8H, t, OCOCH_2_CH_2_N); 2.56 (16H, t, 8(NCH_2_CH_2_OOCNH)); 2.41 (t, 8H, 4(CH_2_OOCCH_2_CH_2_N)); and 1.88 (s, 24H, CH2=C(CH3)CO).

AE2 was prepared using the same components as described for AE1, but adding G-IEMA instead of Bis-GMA (Table 1). Concentration ranges for each component in commercial and experimental adhesives are presented elsewhere [22]. 

The pH of the adhesives was assessed using a high accuracy pH meter Crison Basic20 (Crison Instruments, Barcelona, Spain) (Table 1).

### 2.2. Specimen Preparation

The protocol followed in this study was approved by the Ethics Committee of the Instituto Universitário Egas Moniz, Monte da Caparica, Portugal.

Thirty-two caries-free extracted human molars, stored at 4 °C in 1.0% chloramine T for a maximum of 1 week following extraction, were selected. The roots were cut, and the superficial dentin was exposed under water irrigation by cutting the enamel from the occlusal tooth surface using a hard tissue microtome (Accuton 50, Struers A/S, Ballerup, Denmark). Each dentin surface was ground with 600-grit silicon carbide abrasive paper to produce a standard smear layer. The teeth were then randomly assigned to eight different groups according to the adhesive used, as well as the protocol (SE or ER). The main characteristics of the groups tested are described in Table 1. 

The commercial universal adhesives were then applied in each assigned group, with ER or SE techniques according manufacturer’s instructions. 

The universal adhesives were applied on dentin by mixing a brush tip for 20 s, and gently air blown for 5 s. Polymerization was promoted with a halogen light-curing unit Optilux 501 (Kerr, Middleton, MA, USA) for 10 s. For the etch-and-rinse technique, the dentin surfaces were previous etched with 32% H_3_PO_4_ Scotchbond™ Universal Etchant (3M ESPE, St. Paul, MN, USA) for 15 s, rinsed with water for 15 s and dried carefully with a soft blow of air to leave a moist surface. After each adhesive application, 6–8 mm high resin build-ups were formed in 2 mm increments with a universal light-cured hybrid composite resin—Grandio (VOCO, GmbH, Cuxhaven, Germany). Each layer of the composite was separately light-activated for 40 s according manufacturer’s instructions. Light intensity output was monitored at 600 mW/cm^2^ after every tenth use with a radiometer, Demetron 100 (Demetron Reserach Company, Danbury, CT, USA).

The teeth were identified and stored in distilled water at 37 °C for 24 h in an incubation oven (Memmert INE 400, Memmert, Germany).

### 2.3. Micro-Tensile Bond Strength (µTBS)

After a 24 h storage period, teeth were longitudinally sectioned using a hard tissue microtome (Accuton 50, Struers A/S, Ballerup, Denmark) to yield 1 mm thickness slabs. These slabs were further sectioned into beams with cross-sectional areas of 1 ± 0.3 mm^2^, following the method described by Shono et al. [23]. All beams were attached to a μTBS (micro-Tensile Bond Strength) testing jig with a cyanoacrylate adhesive (Zapit, Dental Ventures of America Inc., Corona, CA, USA) and loaded to failure under tension, using a universal testing machine equipped with a 5 kN loading cell (Shimadzu AG-50kNI SD MS, Shimadzu Corporation, Kyoto, Japan) at a crosshead speed of 0.5 mm/min. The fractured beams were carefully removed from the jig, and the cross-sectional area at the site of failure was measured to the nearest 0.01 mm with a digital caliber (Sylvae Ultra-Call/Fowler Inc., Newton, MA, USA). Fractured specimens were examined with a stereomicroscope (Olympus/DeTrey, Konstanz, Germany) at 40× magnification to determine the mode of failure (adhesive, cohesive or mixed). All the experimental procedures were performed by the same operator in order to reduce random errors.

### 2.4. SEM Analysis 

For each group, two teeth were prepared to be analyzed by SEM (Scanning Electron Microscopy). The dentin surface was exposed, and the adhesives and resin build-ups were applied according to the same procedure described in Section 2.2. The teeth were then stored in distilled water at 37 °C for 24 h. After this period, samples were immersed in a glutaraldehyde and sodium cacodylate fixation solution according to Perdigão et al. [24]. The specimens were sectioned with a hard tissue microtome (Accuton 50, Struers A/S, Ballerup, Denmark) in a perpendicular plan to the adhesive interface, and the un-hybridized dentin was removed after demineralization and deproteinization procedures [25]. They were then dehydrated using increasing concentrations of ethanol (70%, 95% and 100%) and subsequently dried by immersion in methyldisilazane (HMDS) [24]. 

Finally, samples were subjected to a metallization process, where each one was placed on a self-adhesive double-sided carbon tape and then coated with a thin film of approximately 8 nm of Au/Pd on a Q150T ES Turbo-Pumped Sputter coater (Quorum Technologies, Q150T Turbomolecular-pumped coating system, UK). The hybrid layer was then observed at SEM (FEG-SEM JEOL, model JSM7001F, Tokyo, Japan) using 500× and 2000× magnifications. 

### 2.5. Statistical Analysis

In the experimental design implemented in this investigation, the measurement of a variable (adhesive µTBS, in MPa) in different specimens (beams) that originated from the same experimental unit (tooth) was performed. Taking into account that the measures were repeated in systematic way, the effects of possible correlations and heterogeneous variances, introduced by the results obtained in the different specimens, could not be ignored and had to be modeled in the data analysis. 

To control these effects introduced by the repeated measures, the general linear model (GLM) was fitted to the collected data, in which the result of the measurement in the different specimens of the same experimental unit was represented by the mean values obtained in each one of these specimens. Thus, the GLM approach (part A) considered data collected in 32 teeth, considering that each one of these experimental units was represented by the mean of the µTBS calculated in all beams obtained in this tooth. Since they were calculated in different experimental units, these averages were considered independent observations, as required in GLM analysis. 

Another method used to control the effects of the correlations and heterogeneous variance possibly introduced by the repeated measures was a linear mixed model (LMM) fitted to the collected data, which combines the fixed effect of the factors (type of adhesive and protocol) and the random effects in the structure of covariance of the residuals (part B).

Contingency tables and the chi-square test were used to assess the differences in proportions of the observed failures modes, amongst the four adhesives (see Supplementary Information).

## 3. Results

### 3.1. Dentin µTBS 

#### 3.1.1. Part A: GLM Analysis of µTBS Data 

A linear model was fitted to collected data, in which the dependent variable represented the mean value of the dentin sticks µTBS of the 32 teeth, with independent factors defined by the type of adhesive (SBU, FUT, AE1, AE2) and protocol (ER, SE), as presented in Table 2. 

Prior to the analysis, the assumptions of homogeneous variances and normal distribution were tested and validated, using the Levene test (*p* > 0.05) and the Shapiro–Wilk test (*p* > 0.05) respectively. Regarding the normality, the results should be considered with caution, due to the reduced statistical power of the test when applied to small samples.

Under these conditions, the GLM model concluded that:

- There was no significant interaction between the independent factors (adhesives and protocols) (*p* = 0.153).
- There were significant differences between the adhesives (*p* = 0.023), with an observed power of 74.9%.
- Post-hoc tests concluded that AE1 presented a significantly higher µTBS than SBU (*p* = 0.012), with no differences among the other adhesives.
- The ER protocol showed significantly better results compared with SE protocol (*p* = 0.015), with an observed power of 71.0%.

#### 3.1.2. Part B: LMM Analysis of µTBS Data

The use of LMM for the analysis of data collected, with measurements on dentin sticks obtained in the same tooth, requires that the covariance structure between the repeated observations in this experimental unit be known. In the selection of an appropriate and simple covariance structure, comparisons with the unstructured covariance (UN) matrix are usually performed since this represents the most complex covariance structure that can be fitted to the data. However, due to the high number of sticks obtained for each tooth in this study, the UN method did not converge to a solution that guarantees the accuracy of the covariance parameters of the model.

In these conditions, several matrices were considered, and the quality of the adjustment obtained with the different matrices was compared using the Akaike (AIC), Hurvich and Tsai (AICC), Bozdogan (CAIC) and Bayes and Schwarz (BIC) information criteria, which is the most recommended method in view of the wide range of covariance structures considered. Therefore, the selection of the appropriate structure considered matrices for homogeneous variances, namely the Scaled Identity (SI), first-order autoregressive moving average (ARMA(1)), Compound Symmetry (CS) and Toeplitz structures, and matrices for heterogeneous variances, such as the Diagonal, Huynh–Feldt and first-order autoregressive structures (AR(1): Heterogeneous).

According to the various information criteria, particularly the recommended AIC and BIC, and following the accepted interpretation that a smaller value is better, the best adjustment quality of the model was obtained with a covariance structure of compound symmetry (CS) (Table 3). This matrix reflects the existence of homogeneous variances and homogeneous correlations.

Under these conditions, the model obtained allowed us to conclude that:

- No significant interaction was observed between the adhesive and protocol factors (*p* = 0.125).
- The effect of the adhesive type was significant (*p* = 0.010), as well as of the protocol (*p* = 0.019), with better results observed for the ER approach.
- Multiple comparisons showed significant differences only between the AE1 and SBU adhesives (*p* = 0.019).

In conclusion, the results obtained by GLM and LMM analysis were coincident, regarding the magnitude of the effects of the main factors and the lack of interactions.

### 3.2. Failure Modes and SEM Results

Quantitative analysis of the association between failure modes and type of adhesive was performed (Supplementary Information) and produced the following results: 

The prevalence of adhesive fractures in SBU (77.5%) is significantly lower (*p* < 0.001) than in AE2 (91.9%), therefore the prevalence of cohesive fractures in SBU (22.5%) is significantly higher than in AE2 (8.1%).

Moreover, the prevalence of adhesive fractures in AE1 (76.9%) is significantly lower (*p* = 0.001) than in AE2 (91.9%), which means that the prevalence of cohesive fractures in AE1 (23.1%) is significantly higher than in AE2 (8.1%).

In conclusion, differences between adhesives that affect the prevalence of fractures could only be detected between adhesives AE2 and SBU and adhesives AE2 and AE1.

**Figure 1:**
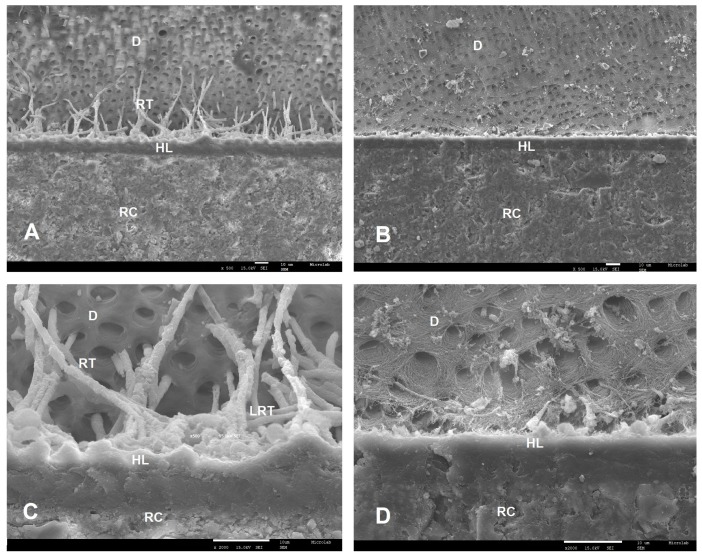
SEM micrographs of bonding interface of the commercial adhesives when ER was applied. Magnifications of 500× (**A**,**B**) and 2000× (**C**,**D**) were used. **A** and **C**—SBU with 11.3 µm of hybrid layer (HL) thickness; **B** and **C**—FUT with 7.6 µm of hybrid layer (HL) thickness. Any resin tags (RT) and lateral resin tags (LRT) were identified. RC—resin composite; D—dentin; HL—hybrid layer; RT—resin tags; LRT—lateral resin tags.

**Figure 2:**
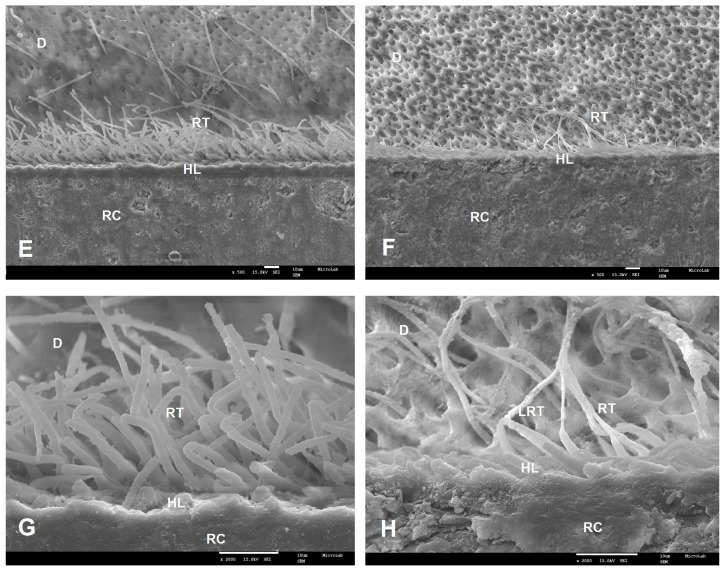
SEM micrographs of the bonding interface of the experimental adhesives when ER was applied. Magnifications of 500× (**E**,**F**) and 2000× (**G**,**H**) were used. **E** and **G**—AE1 with 12.3 µm of HL thickness; **F** and **H**—AE2 with 11.2 µm of HL thickness and resin tags (RT) with 18.2 µm in length. RC—resin composite; D—dentin; HL—hybrid layer; RT—resin tags; LRT—lateral resin tags.

**Figure 3:**
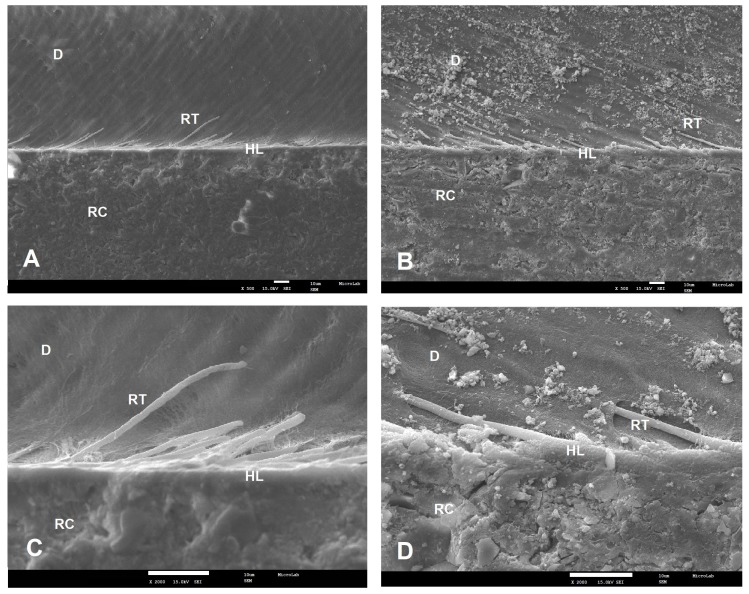
SEM micrographs of the bonding interface of the commercial adhesives when SE was applied. Magnifications of 500× (**A**,**B**) and 2000× (**C**,**D**) were used. **A** and **C**—SBU; **B** and **D**—FUT. RC—resin composite; D—dentin; HL—hybrid layer; RT—resin tags.

**Figure 4:**
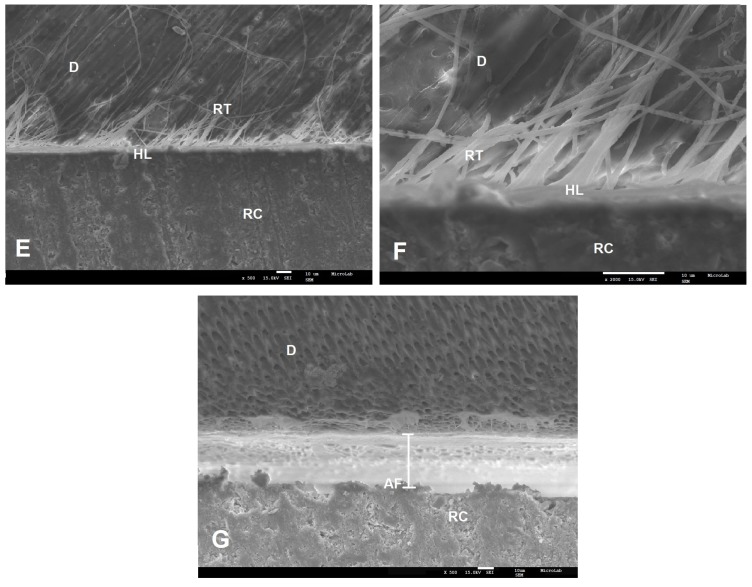
SEM micrographs of bonding interface of the experimental adhesives when SE was applied. Magnification of 500× (**E**,**G**) and 2000× (**F**) were used. **E** and **F**—AE1 with 10.4 µm of HL thickness; (**G**)—AE2. RC—resin composite; D—dentin; HL—hybrid layer; RT—resin tags; AF—adhesive failure.

SEM examinations (magnifications 500× and 2000×) of the resin–dentin interfaces, created by the two commercial universal adhesives (SBU and FUT) and the two experimental universal adhesives (AE1 and AE2), showed different interfacial morphological features in both adhesive protocol, ER and SE (Figure 1, Figure 2, Figure 3 and Figure 4). 

Following ER protocol, a similar morphology of the adhesive interface was observed for SBU, AE1 and AE2. SBU could penetrate the dentin, forming a uniform hybrid layer with 11.3 µm thickness and well-defined resin tags (Figure 1A,C). AE1 and AE2 also showed a uniform hybrid layer with 12.3 µm and 11.2 µm thicknesses, respectively, and a great number of resin tags with different lateral branches (Figure 2E–H). FUT presented the lowest hybrid layer thickness (7.6 µm); in this case, it was not possible to visualize any resin tag or lateral tags (Figure 1B,D).

With the SE approach, SBU and FUT micrographs presented a diffused and undefined hybrid layer with few resin tags (Figure 3A–D). Among all adhesives tested, AE1 presented the best interface morphology. This experimental adhesive seems to penetrate the dentin, forming a hybrid layer with 10.4 µm thickness, with many long resin tags being observed (Figure 4E,F). AE2 micrograph showed an adhesive failure (Figure 4G).

## 4. Discussion

The Micro-tensile Bond Test (μTBS) is considered the most valuable test for the evaluation of the adhesion bond strength [23,26]. This test allows a more uniform stress distribution than the shear bond strength test, due to axial tensile loading on a reduced interface, thus reducing the frequency of cohesive fractures in the dentin [27]. In any case, according to some authors, this test is highly influenced by the procedures and preparation of the samples for testing [26].

It is well known that the performance of the multi-mode adhesives evaluated by in vitro studies depends on the adhesive strategy, and tooth substrate and their efficacy has been reported as material-dependent due to the complexity of their chemical composition [28,29].

According to recently published work, it is consensual that for enamel adhesion, the bond strength of the Universal adhesive is improved when 32–40% phosphoric acid is applied prior to the next stages of dental restoration [7,30]. Regarding dentin adhesion, there is still some controversy among authors [3,30]; however, Sofan et al. [7], referred that adequate bonding can be achieved with either etch-and-rinse (ER) or self-etch protocols (SE).

Concerning the results obtained in this study using GLM and LMM, there was no interaction between the adhesive type and protocol (*p* > 0.05), which means that the observed differences between adhesives concerning their bond strengths are independent of the strategy used to promote dental adhesion.

Independently of the adhesives, samples treated under ER protocol showed significantly higher µTBS than those treated using SE (*p* < 0.05). However, Perdigão et al. [14] defend that SE protocol is more appropriate for dentin when a universal system containing 10-MDP is used. According to these authors, the ER protocol might over-etch dentin structures, decreasing the dentin bond strength due to calcium removal; this consequently decreases the chemical bond strength, which has been referred to as being very important in creating a resistant hybrid layer with more hydrolytic stability, improving the dentin bonding durability [31].

The chemical composition of the adhesives could be the reason for the differences observed in this study, between the AE1 and SBU adhesives, independently of the protocol used. Although AE1 was formulated considering the list of ingredients and their relative percentage range described by the SBU manufacturer, the exact percentage of each component was not disclosed. In addition, unlike AE1, SBU contains the polyalkenoic acid copolymer which, in combination with 10-MDP, has shown contradictory results: Perdigão et al. [32] reported a higher µTBS to dentine for SBU when compared to Clearfil SE Bond, while Muñoz et al. [33] observed a lower µTBS for SBU when compared to the same adhesive. It is noteworthy that Clearfil SE Bond only contains 10-MDP in its composition. It is then possible that the polyalkenoic acid copolymer may compete with the 10-MDP monomer for calcium-bonding in hydroxyapatite [31].

Considering the results obtained for AE1, the dentin µTBS values are within the same range of values of the results published in literature for adhesives containing Bis-GMA. In fact, results similar to those obtained in our study, for both protocols (ER and SE), were reported by Luque-Martinez et al. [34] and Muñoz et al. [5,33], using the same methodology. Moreover, the SEM images of the interface morphology of the adhesives tested are in accordance with the dentin µTBS results, where AE1 presented deeper penetration into dentin tissue with a thicker hybrid layer and long resin tags in both protocols (Figure 2E,G and Figure 4E,F).

Concerning dentin bonding behavior of AE2, it is not possible to establish any type of comparison since we have not found any reports on adhesives systems containing G-IEMA in literature. Furthermore, it is noteworthy that these adhesives are experimental, and further investigation is still needed in order to improve its physicochemical and mechanical properties. However, it should be emphasized that AE2 has shown no significant differences to the other adhesives studied (AE1, SBU and FUT).

A major finding of this work is the achievement of an adhesive Bis-GMA-free, with the same bonding strength properties as other universal adhesives. Both in the health and commercial perspective, this result may represent an important advance in dental materials formulation, since there are no available options in the market of dental adhesives without bisphenol A.

On the other hand, the use of a dendrimer, such as G-IEMA, as the base monomer of the adhesive composition may also bring some physicochemical advantages, as previously reported in our study. In that study, AE2 presented significantly higher double bond conversion, compared to the commercial SBU [22]. A possible explanation was proposed by Yu et al. [20], where G-IEMA allowed the polymerization to occur in eight tips due to its dendritic structure, contributing to a higher double bond conversion rate and greater hydrophobicity. These properties have been considered quite important to the hydrolytic stability of the bonding interface [2,28].

Regarding the pH, this physicochemical property of the UA plays a very important role. On one hand, an acidic medium is needed to dissolve the smear layer and the smear plugs, and open the way for adhesive impregnation into the dentin; on the other hand, a very strong acidic medium might remove too much calcium, consequently decreasing its bonding ability to 10-MDP [35]. Perdigão et al. [14] referred that the ideal pH for UA containing 10-MDP is mild (pH ≈ 2), which allows greater formation of stable calcium salts, improving dentin bonding.

AE2 presents an ultra-mild pH (pH = 3.5). This acidity level does not seem to be sufficient to demineralize the smear layer and to allow the adhesive penetration into the dentin. This fact may possibly explain the results obtained by SEM, where adhesive failures were observed (Figure 4G). It is possible then, that lowering its pH might result in the improvement of its bonding strength properties as a result of a better penetration of G-IEMA’s branches, making it presumably a similar or even a better choice over AE1 (Figure 4F).

On the contrary, in the ER approach, SEM images of AE2 presented a better interface morphology with larger micromechanical retention inside the dentin. The prior application of phosphoric acid in this procedure, which promotes a depth demineralization and the effective penetration of the adhesive, might explain the results. Also important is the fact that G-IEMA has a branched structure and a higher polymerization degree, which may contribute to the entanglement of resin tags (RT) and the formation of many lateral tags (LT). The micromechanical retention effect obtained is stronger. The deeper impregnation of the adhesive, together with its increased polymerization, allowed the formation of a hybrid layer of 11.2 µm in depth, reinforced by the resin tags (Figure 2F,H).

In order to improve the bonding efficacy of AE2, strategies to optimize AE2 composition are currently being investigated.

## 5. Conclusions

Considering the results herein described, the development of a new universal adhesive is still a challenge to adhesive dentistry. The results of this study may represent a paradigm shift in the development of new adhesive systems, which reinforces its originality. It was shown that the traditional linear crosslinking monomers can be replaced successfully by a dendritic type of structures. G-IEMA not only significantly improved the adhesive’s bond conversion rates, but also reduced the polymerization shrinkage and contributed to an increase in dentin bond strength, which lowers the risk of bond failures and subsequent bacteria infiltration that compromise the longevity of the tooth restoration. Nevertheless, further investigation is still being developed in order to improve the physicochemical and mechanical properties of the AE2.

## 6. Patents

The following patent was registered in Portugal as an outcome of this work: Vasconcelos e Cruz, J., Gonçalves, L. L., Polido, M. and Brito, J.A. FORMULAÇÃO PARA UM SISTEMA ADESIVO DENTÁRIO UNIVERSAL CONTENDO UM MONÓMERO DE RETICULAÇÃO DENDRÍTICO DE SEGUNDA GERAÇÃO. “FORMULATION OF A UNIVERSAL DENTAL ADHESIVE SYSTEM CONTAINING A SECOND GENERATION DENDRITIC RETICULATION MONOMER.” (2019) Titular: Instituto Universitário Egas Moniz. Portugal. N. 115064.

## Figures and Tables

**Table 1:** Experimental groups and their respective main components.

Adhesive	Primary Ingredients	pH	Experimental Groups	n	Adhesive System Applied
Scotchbond Universal (3M ESPE, St. Paul, MN, USA), Lot #587502	10-MDP; HEMA; Bis-GMA; ethanol; TEGDMA; silane treated silica; water; CQ	2.95	SBU-ER	5	Etch-and-rinse
			SBU-SE	5	Self-etch
Futurabond M+ (VOCO GmbH, Cuxhaven, Germany), Lot #1624201	HEMA; Bis-GMA; ethanol; Acidic adhesive Monomer; UDMA; pyrogenic silicic acids	1.9	FUT-ER	3	Etch-and-rinse
			FUT-SE	3	Self-etch
AE1 (experimental adhesive formulated with Bis-GMA)	10-MDP; Bis-GMA; HEMA; UDMA; TEGDMA; water; etanol; CQ	2.09	AE1-ER	3	Etch-and-rinse
			AE1-SE	5	Self-etch
AE2 (experimental adhesive formulated with G-IEMA)	10-MDP; G-IEMA; HEMA; UDMA; TEGDMA; water; etanol; CQ	3.5	AE2-ER	5	Etch-and-rinse
			AE2-SE	3	Self-etch

10-MDP: 10-methacryloxydecyl dihydrogen phosphate; HEMA: hydroxyethyl methacrylate; Bis-GMA: bisphenol a diglycidyl ether dimethacrylate; TEGDMA: 1,10-decamethylene glycol dimethacrylate; CQ: camphorquinone, G-IEMA: dendrimer of generation (2) derived from isocyanatoethyl methacrylate; SBU: Scotchbond Universal™); FUT: (Futurabond M+™); ER: Etch & Rinse; SE: Self Etch.

**Table 2:** Mean values and standard deviations (S.D.) of the dentin micro-tensile bond strength (μTBS, in MPa) for the tested groups.

GROUPS	PROTOCOL	µTBS		N
		Mean	S.D.	
SBU-ER	ER	26.20	6.46	5
FUT-ER		33.96	3.22	3
AE1-ER		33.38	6.57	3
AE2-ER		36.50	4.25	5
SBU-SE	SE	23.69	3.15	5
FUT-SE		24.10	2.62	3
AE1-SE		34.66	8.41	5
AE2-SE		25.57	7.48	3

**Table 3:** Information criteria for different covariance structures *.

Information Criteria	Covariance Structures						
	SI	CS	Diagonal	AR(1)	Huynh-Feldt	ARMA(1)	Toeplitz
−2 Restricted Log Likelihood	4278.983	4261.939	4231.212	4278.313	4278.983	4264.345	4228.351
Akaike’s Information Criterion (AIC)	4280.983	4265.939	4289.212	4282.313	4338.983	4270.345	4286.351
Hurvich and Tsai’s Criterion (AICC)	4280.990	4265.962	4292.771	4282.337	4342.794	4270.392	4289.909
Bozdogan’s Criterion (CAIC)	4286.235	4276.442	4441.517	4292.817	4496.540	4286.101	4438.656
Schwarz’s Bayesian Criterion (BIC)	4285.235	4274.442	4412.517	4290.817	4466.540	4283.101	4409.656

* SI: Scaled Identity; ARMA (1): First-order autoregressive moving average; CS: Compound Symmetry; AR (1): First-order autoregressive (Heterogeneous).

## Supplementary Materials

The following are available online at https://www.mdpi.com/2073-4360/12/2/461/s1, Table S1: Cross tabulation with the prevalence of each type of fracture per adhesive.
