# Supplementary material for: Dentin Bonding and SEM Analysis of a New Experimental Universal Adhesive System Containing a Dendrimer
Source: Polymers (Basel). 2020 Feb 17;12(2):461. doi: 10.3390/polym12020461 (PMC7077672; doi:10.3390/polym12020461)
Supplement: Supplementary file 1 [file polymers-12-00461-s001.pdf]

Supplementary Information: Dentin Bonding and SEM Analysis of a New Experimental Universal Adhesive System Containing a Dendrimer

Joana Vasconcelos e Cruz [1,2], Mário Polido [2,3], José Brito [2,3] and Luisa L. Gonçalves [2,3,*]

1 Instituto de Ciências Biomédicas de Abel Salazar (ICBAS), Universidade do Porto, Rua de Jorge Viterbo Ferreira 228, 4050-313 Porto, Portugal; joanavcruz@gmail.com

2 Instituto Universitário Egas Moniz (IUEM), Campus Universitário, Quinta da Granja, Monte de Caparica, 2829-511 Caparica, Portugal; mpolido@egasmoniz.edu.pt (M.P.); britojaa@hotmail.com (J.B.)

3 Centro de Investigação Interdisciplinar Egas Moniz (CiiEM), Campus Universitário, Quinta da Granja, Monte de Caparica, 2829-511 Caparica, Portugal

* Correspondence: lgoncalves@egasmoniz.edu.pt

Received: 26 January 2020; Accepted: 13 February 2020; Published: date

In order to assess whether there is an association between the type of fracture (adhesive (A) or cohesive (C)) and the type of adhesive (SBU, FUT, AE1 and AE2), 2 X 4 contingency tables and the chi-square test were used. Table 4 shows the prevalence of each type of fracture per adhesive. In view of such data, the chi-square test produced a significant result ($p = 0.001$) which shows that there is an association between type of fracture and adhesive. Consequently, 2 × 2 contingency tables and chi-square test with Bonferroni corrections were used to compare the prevalence of adhesive fractures between any two adhesives, with the following results:

-The prevalence of adhesive fractures in SBU (77.5%) is significantly lower ($p < 0.001$) than in 296 AE2 (91.9%), therefore the prevalence of cohesive fractures in SBU (22.5%) is significantly higher 297 than in AE2 (8.1%). 298

- Moreover, the prevalence of adhesive fractures in AE1 (76.9%) is significantly lower ($p = 0.001$) 299 than in AE2 (91.9%), which means that the prevalence of cohesive fractures in AE1 (23.1%) is 300 significantly higher than in AE2 (8.1%).

Table S1. Cross tabulation with the prevalence of each type of fracture per adhesive.

			**Adhesives**				**Total**
			**SBU**	**FUT**	**AE1**	**AE2**	
**Fractures**	**A (adhesive)**	% within adhesive	77.5%	86.1%	76.9%	91.9%	81.8%
	**C (cohesive)**	% within adhesive	22.5%	13.9%	23.1%	8.1%	18.2%
**Total**		% within adhesive	100.0%	100.0%	100.0%	100.0%	100.0%
